# Interweaving Threads: Untangling the Moderating Relationship of Parent-Child Conflict and Closeness in the Association Between Interparental Conflict and Emotion Regulation

**DOI:** 10.1177/21676968241311950

**Published:** 2025-01-04

**Authors:** Katrina R. Abela, Alia Hussain, Danielle M. Law

**Affiliations:** 1 Offord Centre for Child Studies, Department of Psychiatry & Behavioural Neurosciences, 62703 McMaster University, Hamilton, ON, Canada; 2 Department of Psychology, Neuroscience & Behaviour, 62703 McMaster University, Hamilton, ON, Canada; 3 Department of Psychology, 8431 Wilfrid Laurier University, Brantford, ON, Canada

**Keywords:** emotion regulation, parent-child relationship quality, interparental conflict, parenting

## Abstract

The capacity to regulate emotions is central to children’s physical, emotional, and mental well-being as they develop. The influence of adverse childhood experiences on diminished emotion regulation (ER) has been linked to internalizing and externalizing problem behaviours in both children and adolescents. This cross-sectional study, including 479 Canadian emerging adults aged 17–19 years, examined how exposure to different levels of interparental conflict (IPC) during childhood was associated with ER (i.e., expressive suppression and cognitive reappraisal) during emerging adulthood, and how parent-child closeness and parent-child conflict moderated this link. Findings revealed that at higher levels of parent-child closeness, IPC was associated with increased expressive suppression, while there were no significant differences in expressive suppression at lower levels of parent-child closeness. Similarly, IPC was more strongly associated with reduced cognitive reappraisal in the context of high parent-child conflict compared to low conflict. Findings from this work will inform interventional therapeutic and counselling practices to support the well-being of children and families.

## Introduction

The ability to independently regulate emotions is fundamental to healthy development (Compas et al., 2017; Gross, 2002; Thomsen & Lessing, 2020). Emotion regulation (ER), the process of inhibiting or maintaining the experience, intensity, or duration of internal emotional states and/or emotion-related behaviour, is essential for effectively managing emotions and associated behaviours (Denham et al., 2003; Morris et al., 2007). Indeed, current research underscores that fine-tuned ER capabilities serve as an underlying mechanism for shaping development, particularly with respect to problem behaviour, social competence, and overall wellbeing (Compas et al., 2017; Eisenberg & Spinrad, 2004; Morris et al., 2007; Naragon-Gainey et al., 2017; Silk et al., 2003; Thomsen & Lessing, 2020). Notably, children and adolescents who exhibit skillful ER often display less externalizing (e.g., hyperactivity, conduct problems) and internalizing (e.g., anxiety, depression) problem behaviours, higher levels of social competence (e.g., prosocial behaviour), and greater overall physical and psychological wellbeing across the lifespan than those who exhibit ER capacities less skillfully (Eisenberg & Spinrad, 2004; Morris et al., 2007; Thomsen & Lessing, 2020). Crucially, those who demonstrate greater proficiency in ER during childhood exhibit more favorable outcomes than their same-aged peers, across childhood and into adulthood (Zeman et al., 2017).

Over the last several decades, three theoretical models of ER have been proposed, each offering a unique perspective on the mechanisms involved in ER: temporal process models (focusing on the sequence of ER processes and when specific strategies are deployed), strategy-based models (emphasizing the diversity and classification of ER strategies), and ability-based models (highlighting individual differences in ER capacity and regulation across contexts) (Naragon-Gainey et al., 2017). The strategy-based model has garnered considerable attention in developmental psychology due to its focus on specific ER strategies, which provide actionable targets for interventions aimed at enhancing ER skills, particularly during developmental stages marked by transitions and challenges, such as emerging adulthood (Wood et al., 2018). Within this framework, cognitive reappraisal (reframing an interpretation of a potentially emotion-eliciting situation to alter its emotional impact) and expressive suppression (inhibiting outward emotional expressions in response to such situations) (Gross, 1998) have emerged as pivotal strategies for understanding the deployment of ER strategies. Although context-dependent, cognitive reappraisal is generally considered an adaptive ER strategy, whereas expressive suppression is often viewed as maladaptive (see Naragon-Gainey et al., 2017 for a review). Despite their importance, the influence of early family-based experiences on the use of these specific ER strategies remains unclear. Thus, focusing on specific ER strategies may aid in identifying precisely how early family dynamics influence distinct aspects of ER, thereby providing a clearer understanding of developmental outcomes. Given the emotionally demanding transitions during emerging adulthood (e.g., pursuing higher education, entering the workforce, establishing romantic relationships), the use of adaptive ER strategies like cognitive reappraisal is particularly important for navigating these social and emotional demands effectively (Gross, 1998; Wood et al., 2018).

Two factors that independently impact child ER include interparental conflict (IPC) and parent-child relationship quality. IPC is defined as a range of conflict behaviours between parents that can vary in severity and perceived threat, encompassing verbal disagreements (e.g., yelling) to physical aggression (e.g., throwing objects or physical altercations). Additionally, IPC can lead to an array of emotional and psychological outcomes such as elevated perceived threat experienced by children and co-partners, which can vary based on the intensity and resolution of these conflicts (Grych et al., 1992). Parent-child relationship quality encompasses two core elements: closeness and conflict (Morris et al., 2007; Pianta, 1992). Closeness refers to positive aspects of the relationship, such as warmth, responsiveness, and openness, while conflict refers to negative interactions between parent and child (Pianta, 1992). Specifically, greater IPC has yielded poorer ER in children, while the opposite was found for strong parent-child relationships (Modecki et al., 2017; Morris et al., 2007; Naragon-Gainey et al., 2017). Although IPC significantly impacts approximately half of North American children (e.g., ranging from disagreements to yelling and physical altercations; Ottaway, 2010), with many studies documenting broad repercussions such as psychopathologies, adult psychiatric disorders, and perpetual intergenerational violence (Harold & Sellers, 2018; Holmes, 2013), little work has examined the relationship between IPC and ER. By contrast, other work has revealed compelling associations between strong parent-child relationship quality and an array of favourable outcomes, bolstering the development of ER and overall well-being in children (Kerig, 2016). For instance, Nomaguchi (2012) found that caregiver-child dyads with consistently higher levels of closeness experienced increased self-esteem and self-efficacy and decreased internalizing problem behaviours. Conversely, other work has reported a diverse range of deficits linked to high parent-child conflict, such as increased difficulty navigating social relationships, increased feelings of loneliness, irritability and stress, and academic underperformance (Janssens et al., 2021; Kouros et al., 2014; Schiffrin et al., 2013). However, little work has examined the moderating role of the parent-child relationship, and its core facets (i.e., parent-child closeness and parent-child conflict), in the context of ER. Thus, the purpose of the current study was to examine the relationship between IPC and ER, as moderated by parent-child closeness and parent-child conflict.

### Interparental Conflict and its Relationship to ER

Understanding the impact of IPC is an important public health concern due to its prevalence and significant threat to children’s mental health (Cummings & Davies, 2011; Grych & Fincham, 2001). Emotional Security Theory (EST), developed by Davies and Cummings (1994), provides a valuable framework for understanding how and why conflict and hostility between parents are associated with children’s mental health trajectories. EST emphasizes that a key priority for children within emotionally charged situations, such as IPC, is to maintain a sense of safety and security. When children are frequently exposed to intense parental conflicts marked by hostility, violence, and unresolved outcomes, it creates an environment that hinders their ability to achieve emotional security (Morris et al., 2007). In this context, children’s concerns about security in the interparental relationship are thought to stem from an underlying goal system. This system’s functioning can be observed through three main types of response processes: (a) heightened emotional responses, seen in excessive, dysregulated distress during parental conflict; (b) attempts to regulate exposure, which may involve increased avoidance of or direct involvement in parental disputes; and (c) the development of internal beliefs about how parental conflict impacts the well-being of both the child and the family. Prolonged difficulties achieving a sense of safety and security in the interparental relationship are theorized to increase children’s vulnerability to developing ER challenges and psychopathology.

In addition to the direct impact of IPC on children’s ER, IPC may also indirectly affect child ER through a spillover effect involving the parent-child relationship, as documented by a wide array of research (e.g., Erel & Burman, 1995; Sherrill et al., 2017). Marshall et al. (2019) highlight the concept of ‘spillover,’ wherein aggression within the interparental context spills over into parent-child interactions, exacerbating children’s emotional reactions, including heightened fear and withdrawal, which may ultimately weaken the parent-child bond. This spillover can manifest through parental emotional dysregulation, where negative emotional arousal from IPC carries over into interactions with children, leading to heightened irritability and harsh parenting behaviours (Erel & Burman, 1995). Interestingly, spillover effects from IPC do not necessarily preclude the presence of positive relationship qualities within the parent-child relationship. While relatively underexplored in combination, Driscoll and Pianta (2011) emphasize that the parent-child relationship comprises two core dimensions: conflict and closeness, which can coexist to varying degrees. For instance, parent-child closeness may still persist alongside familial conflict, particularly when conflicts are low-level and infrequent. However, it remains unclear whether parent-child closeness is sufficient to buffer children from the adverse effects of IPC exposure. Importantly, these spillover effects illustrate how IPC can have cascading consequences, impacting both the parent-child dynamic and children’s emotional responses to their entire family system.

A child’s ability to regulate their emotions is largely dictated by the shared interactions they experience with, and the observations they make of, their primary caregiver (Meyer et al., 2014). Specifically, in addition to the emotional security of the home, interactions modeled, observed, and shared between and with caregivers, their primary socializing agents, form the basis for children’s ER and play a pivotal role in fostering their development (Meyer et al., 2014). Modelling has long been regarded as a mechanism for children’s learned behaviour. Recent and early work continue to demonstrate a relationship between modelling and children’s ER development. Emde, (1991) found that when children were placed in stressful or novel situations, they tended to respond by socially referencing their parents’ emotion-related reactions. Indeed, how parents interact with one another and respond to their own emotions may inform children’s decisions and emotion-related behaviours, including how to adapt, cope, and manage their own experiences. Moreover, Morris et al., (2007) discovered that parents’ emotional profiles convey insights into the contextual appropriateness of different emotions based on setting, along with strategies for managing these emotions, to their children.

Crucially, the way parents engage with each other significantly influences their child’s ER skills. For instance, if parents frequently exhibit behaviours linked to inadequate ER (i.e., emotional turbulence, heightened reactivity, and physical and vocal expressions such as stomping of feet and yelling), their observant child is more prone to emulate these behaviours in subsequent situations (Thomsen & Lessing, 2020). Conversely, parents who utilize effective ER strategies (e.g., cognitive reappraisal and mindfulness; mindful pauses and controlled breathing, limited impulsivity, and effective communication) in response to emotionally demanding circumstances present their child with opportunities to observe healthy ER in practice, enabling them to learn accordingly (Meyer et al., 2014; Morris et al., 2007).

The integration of EST within a developmental psychopathology lens has been instrumental in elucidating the roles of emotional security and parental ER as mediators between IPC and child adjustment problems. Modelling of parental behaviours, within this framework, plays a critical role in shaping children’s emotional security. Children learn from observing their parents’ emotional reactions, interactions, and emotional expressions, which directly influence their own emotional security and ER development (Cummings & Kouros, 2008). By examining how IPC and parent-child dynamics influence child ER, our research aims to contribute to a more comprehensive understanding of the mechanisms underlying emotional development during emerging adulthood, and the risks posed by high-conflict environments.

In sum, a clear pathway to behavioural maladjustment appears to be through dysregulated emotion processes where children internalize and emulate the maladaptive coping mechanisms of their caregivers (Hajal & Paley, 2020; Meyer et al., 2014; Perry et al., 2020). Thus, impairments to children’s ER can position them at risk for internalizing and externalizing problem behaviours that may compound over time. This framework underscores the need to examine not only the role of IPC, but also the moderating roles of parent-child conflict and closeness in these processes, further highlighting the interconnectedness of familial dynamics in shaping emotional development.

### Moderating Role of Parent-Child Relationships

Scholarship has long documented the psychological benefits of parent-child relationship quality on children’s overall mental health and wellbeing (Morris et al., 2007). A study conducted by Nomaguchi (2012) with children between the ages of 0–22 years revealed that dyads who reported higher parent-child closeness also reported higher self-esteem and self-efficacy, and lower internalizing problem behaviours relative to children who reported lower parent-child closeness. Moreover, a review by Kiel and Kalomiris (2015) found that child ER stemmed directly from the parent-child relationship. Further research on parent-child relationships indicates that, among factors such as parent-child communication, maternal support, and household challenges, the quality of the parent-child relationship consistently mediated the influence of maternal depression on child problem behaviour (Fingerman et al., 2015; McCarty, McMahon & Conduct Problems Prevention Research Group, 2003; Steele & McKinney, 2019). Moreover, it suggests that parent-child closeness can act as a buffer against the negative impacts of stress and adversity, improving overall emotional outcomes.

Alink et al. (2009) found that the parent-child relationship, above and beyond child ER, effectively buffered the ramifications of childhood maltreatment on outcomes for psychopathology in 7–10-year-old children. Moreover, Mullins and Panlilio (2023) revealed that parent-child relationships significantly mitigated the impact of familial violence and trauma symptoms on later development and emotion regulation in their children. Thus, there is evidence to suggest that parent-child relationship quality, specifically parent-child closeness may have main and moderating effects on children’s ER. Perhaps most strikingly, it is evident that parent-child closeness can ameliorate child outcomes, acting as a buffer to the potential detriments of strenuous early childhood experiences. Given this substantial body of evidence, parent-child closeness was selected as one of the focal points of this study to further explore its critical role in moderating the relationship between IPC and child ER during emerging adulthood. The consistent findings of its buffering effects and its proven importance in fostering healthy emotional development underscore the necessity of examining parent-child closeness in this context.

Conversely, high levels of conflict within the parent-child relationship can undermine a child’s emotional security and stability leading to suboptimal outcomes and, plausibly, hindered ER. To this end, other studies have documented a range of risks associated with lower parent-child quality, such as increased internalizing problem behaviours, difficulty navigating romantic relationships during adolescence, and feelings of loneliness, irritability, and stress, across a range of diverse yet interrelated domains (Janssens et al., 2021; Kouros et al., 2014; Schiffrin et al., 2013). Furthermore, previous studies indicate that when EAs perceive their family environment as emotionally expressive and low in conflict, they exhibit better adaptation to the post-secondary environment and enhanced ER (e.g., Cheung et al., 2019; Johnson et al., 2010). Although there is evidence to suggest that parent-child closeness may mitigate the repercussions of adverse childhood experiences on children’s ER development, it remains unclear whether parent-child conflict may exacerbate symptoms of early exposure to IPC, leading to greater deficits in ER. Therefore, parent-child conflict was selected as one of the focal points of this study to further explore its critical role in moderating the relationship between IPC and child ER in emerging adulthood. Examining parent-child conflict within the broader context of family-based adversity is crucial, as it provides a comprehensive understanding of how negative relational dynamics within the family may intensify the adverse effects of IPC on children’s ER during emerging adulthood, as widely documented in the literature.

Indeed, extensive research has focused on unraveling the antecedents, correlates, and outcomes of child exposure to IPC, while concurrently exploring the influence of parent-child relationship quality on development across typical child and youth populations. However, scarce research has incorporated inadequate parent-child relationship quality as a moderating variable, and even fewer studies have examined these constructs within the context of adverse childhood experiences such as IPC or during emerging adulthood. Emerging adulthood (roughly spanning ages 17–25) is a developmental period marked by significant psychological, social, and emotional changes, making it a crucial time to understand lingering effects of early family dynamics (Wood et al., 2018). Understanding ER during emerging adulthood is particularly important, as this developmental stage is characterized by numerous transitions (e.g., leaving the origin home, pursuing post-secondary education, establishing new social relationships), which may make achieving social goals such as social belonging, peer acceptance, and academic success more challenging (McNamara Barry et al., 2016). Consequently, ER skills are crucial for effectively navigating these complex social dynamics. Additionally, ER underpins the attainment of psychological goals that are prominent during this developmental period, including emotional well-being, mental health, and adaptation to novel adult roles – processes that are partly shaped by early family dynamics and depend on functional ER skills (Morris et al., 2007; Wood et al., 2018). Therefore, extending the investigation of IPC and parent-child relationship quality to emerging adulthood fills an important gap in the literature by exploring how early experiences may continue to influence ER into adulthood.

### Purpose

The purpose of the current study is to investigate the association between IPC and children’s ER during emerging adulthood, exploring whether parent-child relationship quality, specifically measures of parent-child closeness and parent-child conflict, moderates this association. Two research questions guide this work: (1) How degree of exposure to IPC during childhood is associated with ER in emerging adulthood; (2) How the strength of parent-child relationships moderates the association between IPC and ER in emerging adulthood. Three hypotheses are explored:

H1a If IPC during childhood is differentially associated with ER in EAs, an inverse relationship between IPC and cognitive reappraisal is expected.

H1b If IPC during childhood is differentially associated with ER in EAs, a positive relationship between IPC and expressive suppression is expected.

H2a If parent-child relationships moderate the association between varying levels of IPC and cognitive reappraisal, then parent-child relationship quality (i.e., parent-child closeness and parent-child conflict, respectively) should significantly moderate the relationship between IPC and cognitive reappraisal. Specifically, it is anticipated that parent-child closeness will buffer, while parent-child conflict will exacerbate the association between IPC and cognitive reappraisal.

H2b If parent-child relationships moderate the association between varying levels of IPC and expressive suppression, then parent-child relationship quality (i.e., parent-child closeness and parent-child conflict, respectively) should significantly moderate the association between IPC and expressive suppression. Specifically, it is anticipated that parent-child closeness will buffer, while parent-child conflict will exacerbate the association between IPC and expressive suppression.

## Methods

### Participants

Upon approval from the university Research Ethics Board (2021; REB #6926), EA participants (herein referred to as ‘participants’) were recruited via the Wilfrid Laurier University Psychology Research Experience Program (PREP) database. Power analysis conducted via G*Power Statistical Consulting determined that a sample size of 150 participants would be sufficient. The initial plan entailed recruiting a community sample via social media platforms to diversify the sample (i.e., Facebook and Instagram), but recruitment challenges were encountered. Consequently, recruitment efforts pivoted to targeting undergraduate EAs using the PREP database. This approach increased accessibility and resulted in a final sample of 479 undergraduate EAs recruited via PREP between the ages of 17 and 19 from Southern Ontario, Canada. This larger sample size, while exceeding the initial target, enhances the robustness and generalizability of the findings. Subjects were invited to participate through a study recruitment advertisement, which included details on the broad objectives of the study (in layman terms), the eligibility criteria, the duration of the study, and compensation for participating (i.e., credit toward an academic course approved by the institution). Participants were eligible for the study if they were between 17 and 19 years of age and grew up with at least one biological or adoptive primary caregiver (e.g., biological parent, biological grandparent, adoptive parent) who was involved in a co-parenting or romantic relationship.

When participants opted into the study, informed consent was attained via Qualtrics prior to proceeding with the questionnaire. Following participation, students were compensated via course credit, as described above. Recruitment efforts yielded an initial 650 responses. Missing data analysis was conducted to determine whether values were missing completely at random (MCAR). Little’s MCAR test (Little, 1988) yielded a non-significant chi-squared value (*p* = .285), indicating that missing values were MCAR. Responses were omitted from the final sample if they were less than 50% complete or duplicates.

After data cleaning, a total of 479 participants were included in the analyses (75% women, 24% men, 0.6% non-binary, 0.2% transgender; *M*_age_ = 18 years). The sample consisted of: 6.9% 17-year-olds, 56.3% 18-year-olds, and 36.8% 19-year-olds. The self-identified ethnic and racial composition included: 60% White, 25% Asian or Asian Canadian, and 10% Black. Moreover, participants provided information about their caregivers’ co-parenting and living situations throughout their childhood (i.e., between the ages of 5 and 12). The data revealed that 82.7% of caregivers were married or together throughout childhood, 11.7% had joint or shared custody, 5% had sole custody, and .6% had another unspecified arrangement.

The caregivers’ educational attainment composition, as identified by participants, included: 3% Some High School, 13.5% High School, 25.2% College, 2.7% Technical/Professional School, 31.9% Bachelor’s, 18.8% Master’s, 1.3% Doctorate, 1.7% Medical or Juris Doctorate, and 1.9% Other. The caregivers’ yearly income, as identified by participants, included: 19.5% with incomes lower than 50,000 CAD, 31.4% with incomes between 50,000 CAD and 99,999 CAD, 33.2% with incomes 100,000 CAD and higher, and 20.5% Unknown.

### Measures

Self-report questionnaires were administered electronically via Qualtrics. Participants completed the questionnaire independently using their own device (e.g., laptop, mobile phone, tablet) and access to the internet.

#### Demographic Information

Participants were asked to complete a demographic and biographic questionnaire about themselves and their primary caregiver. This questionnaire included items on gender, age, ethnicity, family composition, parental relationship status (i.e., whether the parents are together or separated), details of co-parenting arrangements or child custody (e.g., joint custody, sole custody, visitation schedules), and the highest level of educational attainment in the household.

#### Degree of Interparental Conflict

The Children’s Perception of Interparental Conflict scale (CPIC) (Grych et al., 1992) was used to assess perceptions of IPC on several dimensions of interparental and marital conflict, as well as degree of adjustment and adaptability. The standard measure includes 51 *True*, *Sort of true*, or *False* response items dispersed across nine subscales and three superordinate scales: Conflict Properties (i.e., frequency, intensity, resolution; e.g., *“I often saw or heard my caregivers arguing”*), Threat (i.e., perceived threat, coping efficacy; e.g.*, “When my caregivers argued I felt afraid that something bad might happen”)*, and Self-Blame (i.e., content, self-blame; e.g., *“My caregivers would often get into arguments when I did something wrong”*). A higher score indicated greater conflict. Participants were asked to reflect and report on exposure to IPC during a specified timeframe in their childhood (i.e., *“…we would like you to reflect on your childhood [*i.e.*, when you were between 5-12 years of age] and write what you thought or felt when your caregivers argued by answering each of the sentences below”*). For the purpose of this study, only the Conflict Properties and Threat superordinate scales (27 items) were included in the analysis. The selection of the Conflict Properties and Threat subscales was guided by the research objectives, which focused on the observable and perceived dimensions of IPC. While the Threat subscale captures the child’s subjective interpretation of conflict, it directly relates to the perceived severity and potential impact of IPC, critical for understanding the EAs emotional response to conflict exposure. Conversely, the Self-Blame subscale centers on the child’s attribution of responsibility for the conflict, which extends beyond the aim to examine the conflict characteristics and perceived threat. Including the Threat subscale aligns with the objective to assess both the external and perceived internal dimensions of IPC, enhancing the understanding of how EAs experience, and are affected by IPC. This approach captures a comprehensive picture of the impact of IPC without conflating it with the EAs sense of personal responsibility for the conflict. A composite score for IPC was obtained by calculating the sum scores across the two subscales (Conflict Properties and Threat), as instructed by Grych and colleagues (1992). Consistent with previous studies, our reliability was high: Conflict Properties (*a =* .94), Threat (*a* = .87), and overall CPIC (all 27 items; *a =* .95).

#### Emotion regulation

Emotion regulation (ER) was gauged using the Emotion Regulation Questionnaire for Children and Adolescents (ERQ-CA) (Gross & John, 2003), a widely endorsed measure designed to assess how frequently children and youth employ two regulatory strategies – cognitive reappraisal and expressive suppression – in response to emotionally demanding circumstances on a daily basis. The Cognitive Reappraisal and Expressive Suppression subscales of the ERQ-CA have been reported to have moderate validity against the Children’s Depression Inventory (*r*s = −.26 and .37, respectively; Gullone & Taffe, 2012). The ERQ-CA comprises 10 items (6 assessing Cognitive Reappraisal, e.g., “*When I want to feel happier, I think about something different*,” and 4 evaluating Expressive Suppression, e.g., “*I keep my feelings to myself*”) on a 7-point Likert response scale ranging from 1 (Strongly disagree) to 7 (Strongly agree), where higher scores represent greater employment of the ER strategy in EAs. A composite score was derived independently for each strategy by calculating the sum totals as instructed by Gross and John (2003). Our tests yielded high internal consistency across the Expressive Suppression (*a* = .80) and Cognitive Reappraisal (*a* = .85) scales, echoing past reports.

#### Parent-Child Relationship Quality

The Child-Parent Relationship Scale (CPRS; Pianta, 1992) is a parent self-report questionnaire that included 30 items on a five-point Likert scale ranging from 1 (*Definitely does not apply*) to 5 (*Definitely applies)*, and covered three distinct constructs: Conflicts, Closeness- Positive Aspects of the Relationship, and Dependence. Participants were asked to reflect and report on the quality of the relationship shared with whoever they considered their primary caregiver during childhood (i.e., “*Please reflect on your relationship with your parent during childhood [when you were 5 to 12 years old]. To what degree would each of the following statements apply to you?”*). The Conflict subscale includes items such as *“My parent and I always seemed to be struggling with each other*”, while the Closeness subscale includes items like *“My parent made me feel important and special”*. The CPRS has been reported to have good construct validity against the Family Assessment Device (*r* = .35 and .46; Wamboldt et al., 2001). A composite score was derived for *Conflicts* and *Closeness*, by calculating the mean score across item responses, consistent with the instructions of Pianta (1992). Though the measure was designed to measure caregiver self-report of 3- to 15-year-old children, for this study, the measure was adapted for EA self-report as the item wording was integral to exploring participants’ retrospective perceptions of childrearing, parent-child conflict, parental support and engagement, and parental attitudes, behaviours, and feelings toward them. However, it is important to note that the Dependence subscale was omitted as the original items (e.g., “*My child appears hurt or embarrassed when I correct him/her”*) were constructed to assess child over-dependence on the parent and could not be appropriately adapted to our sample of EAs without compromising the psychometric integrity of the scale (i.e., we would need to rewrite the entire item, given that the dependence items were context-specific to a parenting perspective and do not easily translate to an EA self-report format). Our study revealed adequate internal consistency across both Conflict (*a* = .75) and Closeness - Positive Aspects of the Relationship (*a* = .86) subscales, respectively.

#### Control Variables

Caregiver education and income were assessed using separate items for caregiver 1 and caregiver 2. Caregiver education was rated on a 9-point scale ranging from 1 (some high school) to 9 (medical doctor, doctorate, juris doctor), while caregiver income was rated on a 6-point scale ranging from 1 ($0 - $24,999 a year) to 6 ($150,000+ a year).

### Data Analysis

Listwise deletion was employed for duplicates and cases missing more than 50% of data. Preliminary analyses were conducted to check for violations of assumptions for normality, linearity, homoscedasticity, and multicollinearity. Descriptive statistics and bivariate correlations were conducted for the study variables. Linear regression and moderation analyses were used to investigate the pattern of associations among multiple variables, explore main effects, and compare which variables shared stronger or weaker associations with the outcome variable (Menard, 2010). We controlled for caregiver education and income because of their documented associations with IPC and ER (Dereli, 2016; Meredith & Silvers, 2024; Shaffer et al., 2012). Specifically, caregiver education and income for participants’ primary caregivers were entered into the analyses as covariates as responses to the CPRS were anchored to whoever participants viewed as their primary caregivers during childhood. To address the research objectives, linear regression with moderation analyses, using Hayes’, (2017), was employed to examine how degree of IPC exposure during childhood was associated with ER capacities (i.e., cognitive reappraisal and expressive suppression) in EAs after controlling for parental income and education (hypothesis 1). The independent (i.e., IPC) and moderating (i.e., parent-child closeness and conflict) variables were mean-centered and inputted into moderation analyses to investigate how parent-child closeness and conflict influenced the association between IPC and each ER capacity (hypothesis 2). To further examine significant two-way interactions, the associations between the independent (i.e., IPC) and dependent (i.e., ER capacities) were explored at high (+1 SD) and low (−1 SD) levels of the moderators.

## Results

Descriptive statistics and bivariate correlations among study variables were examined and entered into Table 1. Findings indicated that IPC was significantly and negatively correlated with cognitive reappraisal, expressive suppression, and parent-child closeness. Contrastingly, higher IPC was significantly associated with higher parent-child conflict. Moreover, findings revealed that high levels of cognitive reappraisal were associated with high levels of expressive suppression and parent-child closeness but did not share a significant linear relationship with parent-child conflict. Expressive suppression was significantly and negatively correlated with parent-child closeness and conflict. The analyses also showed that low levels of parent-child closeness were significantly associated with high levels of parent-child conflict. Notably, only 2% of the sample yielded a CPIC score of 67 or above, indicating extreme and chronic exposure to IPC during childhood that substantially impacts the child’s well-being (Grych & Fincham, 2001; Kitzmann et al., 2003).

**Table 1.** Descriptive Statistics and Correlations for Study Variables.

Variable	*n*	*M* (*SD*)	Min	Max	Range	Skew	Kurtosis	1	2	3	4	5
1. IPC	449	44.48 (14.16)	27.00	81.00	35.00–72.00	.21	−.39	-				
2. Cognitive reappraisal	474	28.36 (6.49)	6.00	42.00	6.00–42.00	−.09	−.15	−.10*	-			
3. Expressive suppression	479	16.48 (5.44)	4.00	28.00	4.00–28.00	−.17	−.49	−.19**	.12**	-		
4. Parent-child closeness	473	3.86 (1.96)	1.00	5.00	1.00–5.00	−.87	−.07	−.56**	.16**	−.31**	-	
5. Parent-child conflict	475	2.58 (1.06)	1.00	5.00	1.00–5.00	.36	−.59	.59**	−.08	.29**	−.22**	-

*Note.* IPC denotes interparental conflict. **. Correlation is significant at the .01 level (2-tailed). *. Correlation is significant at the .05 level (2-tailed).

### Interparental Conflict and ER

The current study utilized Hayes’ PROCESS macro, employing moderation analysis through linear regression to examine how degree of IPC exposure was associated with EAs’ ER after controlling for the influence of socioeconomic status and parent education. Moderation analyses explored how parent-child closeness and conflict modified the nature (i.e., strength and direction) of the relationship between IPC and ER. Preliminary analyses revealed no violation of assumptions for normality, linearity, homoscedasticity, and multicollinearity; thus, it was deemed safe to proceed (see Table 1).

The degree of IPC was not significantly associated with either ER strategy (i.e., cognitive reappraisal, *β* = −.010, *p* = .586; expressive suppression, *β* = .004, *p* = .640; see Table 2) in EAs.

**Table 2.** Regressions Examining the Main Effect of IPC and the Moderating Effects of Parent-Child Closeness and Conflict.

Variables	*B*	Std. β	SE	*t*	R^2^	F
Cognitive Reappraisal (*n* = 469)						
Intercept	24.068***	28.010***	.340	82.33	.032*	2.24
IPC	.092*	−.010	.011	−.09		
Parent-child closeness	.258	.202***	.058	3.47		
IPC x parent-child closeness	−.001	−.008	.002	−.46		
Parent-child conflict	.303*	−.051	.050	−1.02		
IPC x parent-child conflict	−.005*	−.005*	.003	−1.97		
Caregiver income	.021	.021	.032	.65		
Caregiver educational attainment	−.028	−.028	.027	−1.03		
Expressive suppression (*n* = 469)						
Intercept	24.39***	16.506***	.275	60.13	.010***	7.14
IPC	−.018	.004	.009	.47		
Parent-child closeness	−.447***	−.218***	.047	−4.66		
IPC x parent-child closeness	.003*	.003*	.002	2.31		
Parent-child conflict	.152	−.116*	.040	−2.90		
IPC x parent-child conflict	−.004	−.004	.002	−1.86		
Caregiver income	.006	.006	.026	.23		
Caregiver educational attainment	−.015	−.015	.022	−.69		

*Note.* IPC denotes interparental conflict. ***. Correlation is significant at the .001 level (2-tailed). *. Correlation is significant at the .05 level (2-tailed).

### The Moderating Role of Parent-Child Relationships

**Figure 1.**
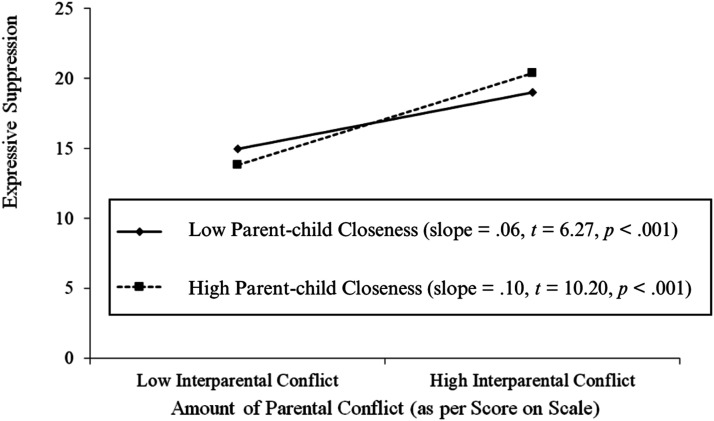
Regression slopes examining the relationship between interparental conflict (IPC) and expressive suppression at high and low levels of parent-child Closeness. *Note.* *p < .05. **p < .01 ***p < .001.

To investigate how parent-child relationships might moderate the nature of the relationship between IPC and the two ER strategies (i.e., cognitive reappraisal and expressive suppression) in EAs, parent-child closeness and parent-child conflict were inputted into the models as moderators. Both regression models demonstrated an excellent fit (cognitive reappraisal, *R*^*2*^ = .032, *F* (7, 469) = 2.24, *p* = .030; expressive suppression, *R*^*2*^ = .010, *F* (7, 469) = 7.14, *p* < .001). Findings revealed an interaction between parent-child closeness and IPC in that parent-child closeness significantly moderated the relationship between IPC and expressive suppression (*β* = .003, *p* = .021; see Figure 1). To examine the moderating role of parent-child conflict on the relationship between IPC and expressive suppression, moderators were entered as was performed for parent-child closeness. Here, findings revealed that parent-child conflict did not significantly moderate the link between IPC and expressive suppression (*β* = −.004, *p* = .06; see Table 2). The moderating effect of parent-child closeness was further examined. Specifically, it was found that the relationship between IPC and expressive suppression was significant at low and high levels of parent-child closeness (see Figure 1).

**Figure 2.**
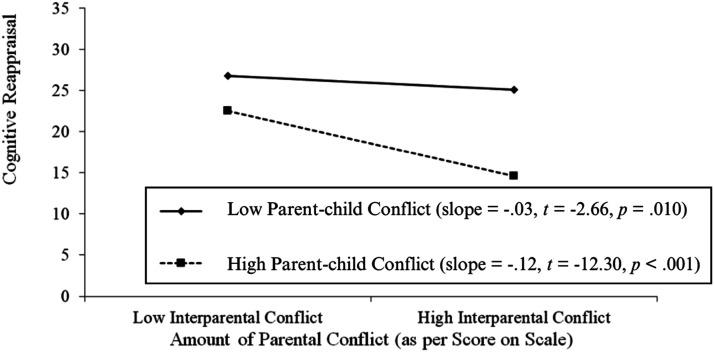
Regression slopes examining of the relationship between interparental conflict (IPC) and cognitive reappraisal at low and high levels of parent-child conflict.

The same analyses were applied to investigate the moderating role of parent-child closeness on the relationship between IPC and cognitive reappraisal, which was found to be non-significant (*β* = −.008, *p* = .65; see Table 2). However, findings indicated that parent-child conflict was indeed a significant moderator of the association between these IPC and cognitive reappraisal (*β* = −.005, *p* = .049; see Table 2). Regression slopes were computed to examine the moderating effect of parent-child conflict. As shown in Table 2, parent-child conflict significantly moderated the association between IPC and EAs’ cognitive reappraisal. Notably, it was found that the relationship between IPC and cognitive reappraisal was significant at low and high levels of parent-child conflict (see Figure 2).

## Discussion

The current study aimed to examine how exposure to IPC during childhood was associated with ER in emerging adulthood, specifically focusing on two ER strategies: cognitive reappraisal and expressive suppression. Furthermore, this study explored the moderating roles of two distinct aspects of parent-child relationship quality – closeness and conflict – on these associations. Our findings indicate that each dimension of parent-child relationship quality significantly moderated the association between IPC and specific ER strategies. Notably, greater IPC exposure was associated with increased use of expressive suppression, particularly when parent-child closeness was low, and decreased use of cognitive reappraisal, especially when parent-child conflict was high. These findings underscore the complex interplay between IPC and parent-child relationship quality in influencing ER outcomes, providing important insights into how early family dynamics contribute to ER during emerging adulthood. This study is one of the first to analyze IPC, parent-child conflict, and parent-child closeness, along with two distinct ER strategies in a single model with a robust sample of 479 EAs. Furthermore, by adopting a strategy-based approach rather than relying on a global score of ER, we were able to highlight nuanced, specific associations between early adversity and two specific ER strategies. These contributions have important implications for designing targeted interventions aimed at fostering adaptive ER strategies in at-risk populations, thereby enhancing developmental outcomes.

### Interparental Conflict and ER

The anticipated significant associations between IPC, cognitive reappraisal, and expressive suppression were not found in our study. One potential explanation is the composition of our sample: only 2% of participants reported extreme exposure to IPC, while the majority reported low or average levels of conflict. This underrepresentation of high IPC may have reduced our ability to detect meaningful effects. Furthermore, based on EST (Davies & Cummings, 1994), children exposed to higher levels of parental conflict may struggle with emotional security, leading to dysregulated responses rather than the deliberate engagement in emotion regulation strategies. This discrepancy could indicate that in higher-conflict environments, children might not have had opportunities to learn adaptive ER strategies, depending on the way conflict was navigated. For instance, when conflict was expressed through avoidance or disengagement (e.g., a parent using the silent treatment), children may have been left without observable coping behaviours to model, which can explain the lack of significant associations in our findings (Cummings & Davies, 2011; Harold & Sellers, 2018).

A second possible explanation concerns the challenges associated with observing ER processes, such as emotional inhibition, modulation, and strategy deployment, which could explain the lack of significant associations found in our study. Effective parental modelling of adaptive ER strategies, such as cognitive reappraisal, is crucial for providing children with a framework to learn and apply these skills effectively (Morris et al., 2007). However, when parents do not explicitly demonstrate or coach adaptive ER behaviours, children may struggle to internalize these strategies, potentially resorting to inconsistent or unproductive coping responses. Our study did not include measures of unproductive coping responses nor emotion-related parenting behaviours such as emotion coaching or modelling, which may have provided valuable insights into how IPC impacts ER development through parental influence (Meyer et al., 2014). Additionally, given that ER strategies like expressive suppression and reappraisal are often internally driven, the absence of observable parental cues may have contributed to the lack of significant associations (Morris et al., 2007).

Lastly, individual differences in emotional reactivity and sensitivity may also play a critical role in the outcomes observed in this study. Children differ widely in their susceptibility to environmental stressors, with some being more vulnerable to the negative effects of IPC than others (Belsky & Pluess, 2009). This variation could explain why the anticipated associations between IPC and ER strategies were not uniformly observed in the current sample. Research has suggested that certain individuals exhibit heightened biological or psychological sensitivity, which may make them more susceptible to either thriving or struggling depending on the level of parental support or conflict they experience (Ellis & Boyce, 2008). Accounting for these individual differences in future studies may help to clarify the conditions under which IPC significantly impacts ER.

### The Moderating Role of Parent-Child Relationships

The moderation analyses provided insights into how two aspects of parent-child relationship quality – parent-child closeness and parent-child conflict – moderated the relationship between IPC and ER strategies (cognitive reappraisal and expressive suppression) during emerging adulthood. It was hypothesized that parent-child closeness would buffer the impact of IPC on ER, promoting greater use of adaptive ER strategies like cognitive reappraisal while reducing the reliance on expressive suppression. Conversely, it was expected that parent-child conflict would exacerbate the negative impact of IPC, leading to lower use of cognitive reappraisal and increased use of expressive suppression. These hypotheses were partially supported, as parent-child closeness and conflict moderated the relationship between IPC and each ER strategy, although some of the effects were more complex than initially anticipated.

Greater parent-child closeness during childhood moderated the relationship between IPC and expressive suppression, but not in the expected direction. Specifically, greater IPC was associated with greater use of expressive suppression at both low and high parent-child closeness, though the relationship was somewhat stronger when parent-child closeness was high. This finding suggests that high parent-child closeness, in the context of elevated IPC, may contribute to some vulnerability, where children suppress emotions potentially to protect the parent or maintain family harmony. This could reflect a dynamic where children adopt suppressive behaviours to shield their caregivers from emotional distress – a phenomenon known as “parentification” (Byng-Hall, 2002; Van Parys & Rober, 2013). In this context, high closeness may foster a sense of responsibility in the child to protect their caregiver, prompting them to inhibit emotional expression. Alternatively, children may suppress emotions as a form of self-protection (i.e., a safety behaviour), fearing that emotional expression during conflict could escalate the situation or redirect the conflict toward them (Erel & Burman, 1995; Sherrill et al., 2017). This response may be particularly relevant when the parent they typically rely on for emotional support is involved in the conflict, rendering them unable to assist the child in managing emotions stemming from witnessing the conflict. Therefore, this suggests that the interaction between IPC and parent-child closeness is complex, where closeness may promote adaptive ER under some circumstances but lead to suppression in others, particularly when IPC is high.

Parent-child conflict, as a moderator, also significantly influenced ER outcomes, particularly in the context of cognitive reappraisal. The findings showed that greater exposure to IPC was associated with less use of cognitive reappraisal at both low and high levels of parent-child conflict. However, the relationship was stronger when parent-child conflict was high. This suggests that a lower-conflict parent-child relationship can foster adaptive coping strategies even amidst high IPC exposure. This finding may extend Emotional Security Theory (EST; Davies & Cummings, 1994) by emphasizing that lower parent-child conflict provides an emotionally secure environment, potentially restoring a child’s sense of security that IPC might undermine (Cummings & Davies, 2011; Harold & Sellers, 2018). Moreover, this outcome aligns with Morris et al.'s (2007) Tripartite Model of Familial Influence, which designates the parent-child relationship as a primary socializing agent of children’s ER.

In contrast, individuals exposed to both higher IPC and higher parent-child conflict demonstrated lower use of cognitive reappraisal. Persistent exposure to both interparental and parent-child conflict may undermine emotional security, making it challenging for individuals to effectively utilize adaptive strategies such as cognitive reappraisal (Davies & Cummings, 1994; Cummings & Davies, 2011). According to the spillover hypothesis, negative emotions in the interparental context can spill over into parent-child interactions, increasing stress and reducing the opportunities for children to develop adaptive ER strategies (Erel & Burman, 1995; Sherrill et al., 2017). Furthermore, higher conflict within the parent-child relationship may limit parents’ capacity to provide effective emotion coaching, diminishing the child’s ability to learn adaptive coping mechanisms (Meyer et al., 2014; Morris et al., 2007). This emphasizes the cumulative impact of multiple sources of conflict on a child’s capacity to regulate emotions adaptively, thereby highlighting the interplay of relational contexts on emotional development and offering insights into potential points for intervention.

### Limitations

This study has several limitations that warrant consideration when interpreting the findings. First, the cross-sectional design limits the ability to infer causal relationships between IPC, ER, and parent-child dynamics. While the findings provide important insights into these associations, longitudinal methods are necessary to explore the long-term effects of IPC on the development of ER strategies over time.

Second, while reliance on retrospective self-reports introduces potential recall bias, these self-reports allow participants to provide personal insights into past experiences with IPC that may not be observable through other means. However, future studies would benefit from integrating multiple sources of data, such as observational and physiological methods, to complement self-reports and provide a more comprehensive picture of how IPC relates to ER. Moreover, although this study focused on two key ER strategies – cognitive reappraisal and expressive suppression – our exploration was not exhaustive. Other important ER strategies, such as avoidance, acceptance, and rumination, were not assessed. Future studies should explore a broader range of both adaptive and maladaptive ER strategies to offer a more complete understanding of ER in children and emerging adults.

Third, variability in caregiving arrangements (e.g., nuclear families, joint custody, or sole custody with a co-caregiver) and potential changes in custody status over the seven-year age span (e.g., transitioning from married to divorced) may introduce measurement error in the IPC construct. While IPC was anchored to the primary caregiver, the use of a true/false response format, despite using a standardized scale (Grych et al., 1992), may not fully capture the complexity of conflict in diverse caregiving contexts.

Finally, our sample consisted primarily of female, college-aged participants, which limits the generalizability of the findings. This demographic homogeneity may not reflect the broader population of EAs, especially those with different socioeconomic, familial, or educational backgrounds. Relatedly, the limited variability in IPC exposure within the sample prevents a thorough examination of the effects of more severe forms of interparental conflict on ER development. Future studies should aim to include more diverse sample populations, incorporating individuals from various backgrounds to provide a more comprehensive understanding of how IPC is associated with ER across different contexts.

### Implications and Future Directions

This study makes important contributions to clinical practice and theoretical understanding. Firstly, it marks one of the first to comprehensively analyze the association between IPC and specific ER strategies, accounting for the potential moderating effect of parent-child closeness and parent-child conflict, respectively. It yielded findings to support the advocacy for ER training approaches aimed at instilling *specific* strategies for children and EAs to emotionally regulate through preventive interventions. While previous research, including recent meta-analyses (e.g., Eadeh et al., 2021; Moltrecht et al., 2021), has demonstrated the effectiveness of psychological interventions in improving ER and reducing pathology symptoms in EAs, our study emphasizes the importance of considering the interconnectedness of ER between parents and children when developing ER-oriented preventative interventions and has illustrated how these important familial dynamics operate with and against each other.

Many studies examining the association between IPC and ER analyze ER as a global construct, neglecting to capture nuance by subscribing to the strategy-based model, and thus, operationalization of ER. Similarly, evidence-based interventions may neglect to explicitly target ER, and those that do, such as emotion-focused cognitive-behavioral therapy (CBT), emotion regulation training, and third-wave interventions (e.g., mindfulness-based therapies), typically focus on EAs (i.e., offspring) or adults (i.e., caregivers) independently (Eadeh et al., 2021). However, our findings suggest that parental and child ER are much more intertwined than previously suspected, particularly in the context of IPC and parent-child dynamics. These dynamics may have significant implications for EAs’ ER and especially among those considered at-risk. Therefore, preventative intervention efforts should aim to bridge ER training for EAs and adults to be more dyadic in nature. Scientists and clinicians can simultaneously introduce EAs at-risk and their parents to specific strategies (e.g., enhancing cognitive reappraisal, reducing expressive suppression) to help them cope with stress and emotional reactivity, thereby fostering an ER “toolbox.”

Alternatively, therapeutic approaches may leverage ER in family contexts, targeting the ER repertoire of parents of young children, and better equipping them with the skills necessary to effectively scaffold their child’s emotional learning. By factoring in the role of parents and their ability to regulate emotions amid conflicts and parent-child interactions, we can develop more comprehensive and effective ER interventions. This dual focus on both individual and familial interventions could potentially enhance the overall effectiveness of ER strategies in preventing emotional and behavioural issues in at-risk EAs and earlier.

Future research should employ RCTs and longitudinal methods to track how child ER varies as a function of IPC and parent-child dynamics across development. By highlighting the need for interventions that address both parent and child ER, our findings contribute to a more comprehensive understanding of how to foster effective ER skills in children exposed to IPC. Studies should also continue to strengthen the understanding of the relationship between adverse childhood experiences and various ER strategies in childhood and emerging adulthood. By narrowing our focus to examine specific ER strategies and the emergence of ER repertoires, we may gain deeper insight into which ER strategies are most successful in reducing stress reactivity and preventing psychopathology in both at-risk and healthy child and EA populations. This knowledge is imperative for informing clinical practice, enabling clinicians and scientists to bolster specific ER strategies through preventative intervention. This approach may tailor therapeutic practices to better meet the behavioural needs of children and eventual EAs.

## Conclusion

In summary, this study explored the differential associations between childhood exposure to IPC and children’s ER during emerging adulthood, along with the moderating role of parent-child relationship quality. While the anticipated associations between IPC and ER were not fully substantiated, insightful patterns emerged. The moderation analyses revealed novel findings, indicating that greater parent-child closeness, particularly in contexts of high IPC, was associated with increased use of expressive suppression, suggesting some vulnerability. Similarly, individuals with higher parent-child conflict showed a stronger negative association between IPC and cognitive reappraisal, indicating that higher conflict exacerbated the reduction in reappraisal strategies as IPC increased. These findings underscore the complex interplay between parent-child relationship quality, IPC, and children’s ER strategies, pointing towards the potential efficacy of preventative intervention targeting parent-child conflict in mitigating maladaptive ER tendencies in children and eventual EAs. Furthermore, we predict, based on the patten of statistically significant and trend-level findings in the current research, that negative emotion-related behaviours, such as parent-child conflict, may exert a stronger influence on child outcomes relative to positively-valence behaviours such as parent-child closeness.

## Supplemental Material

Supplemental Material - Interweaving Threads: Untangling the Moderating Relationship of Parent-Child Conflict and Closeness in the Association Between Interparental Conflict and Emotion Regulation Supplemental Material for Interweaving Threads: Untangling the Moderating Relationship of Parent-Child Conflict and Closeness in the Association Between Interparental Conflict and Emotion Regulation by Katrina R. Abela, Alia Hussain, and Danielle M. Law in Emerging Adulthood.
